# Quantify the Protein–Protein Interaction Effects on Adsorption Related Lubricating Behaviors of α-Amylase on a Glass Surface

**DOI:** 10.3390/polym12081658

**Published:** 2020-07-25

**Authors:** Nareshkumar Baskaran, You-Cheng Chang, Chia-Hua Chang, Shun-Kai Hung, Chuan-Tse Kao, Yang Wei

**Affiliations:** Department of Chemical Engineering and Biotechnology, National Taipei University of Technology, 1, Section 3, Zhongxiao East Road, Taipei 10608, Taiwan; adamnaresh1818@gmail.com (N.B.); youchengchang@gmail.com (Y.-C.C.); t104320088@ntut.org.tw (C.-H.C.); t105320036@ntut.org.tw (S.-K.H.); chuantsekao@gmail.com (C.-T.K.)

**Keywords:** dental ceramic material, glass surface, α-amylase, protein–protein interactions, conformational changes, friction coefficient

## Abstract

Dental ceramic material is one of the widely preferred restorative materials to mimic the natural tooth enamel surface. However, it has continuously been degraded because of low wear resistance during mastication in the oral cavity. The friction involved was reduced by introducing the lubricant saliva protein layers to improve the wear resistance of the dental materials. However, little is understood regarding how the protein–protein interactions (PPI) influence the adsorbed-state structures and lubricating behaviors of saliva proteins on the ceramic material surface. The objective of this study is to quantify the influences of PPI effects on the structural changes and corresponding oral lubrications of adsorbed α-amylase, one of the abundant proteins in the saliva, on the dental ceramic material with glass as a model surface. α-Amylase was first adsorbed to glass surface under varying protein solution concentrations to saturate the surface to vary the PPI effects over a wide range. The areal density of the adsorbed protein was measured as an indicator of the level of PPI effects within the layer, and these values were then correlated with the measurements of the adsorbed protein’s secondary structure and corresponding friction coefficient. The decreased friction coefficient value was an indicator of the lubricated surfaces with higher wear resistance. Our results indicate that PPI effects help stabilize the structure of α-amylase adsorbed on glass, and the correlation observed between the friction coefficient and the conformational state of adsorbed α-amylase was apparent. This study thus provides new molecular-level insights into how PPI influences the structure and lubricating behaviors of adsorbed protein, which is critical for the innovations of dental ceramic material designs with improved wear resistance.

## 1. Introduction

Human teeth are an essential facial aesthetic organ in the mouth [1], but the missing tooth problem is expected to grow over the next two decades because of the aging population [2]. In dentistry, for the restoration of function and esthetics of a defective or missing natural tooth, ceramic materials are widely preferred by both dentists and patients because of its most aesthetic, biocompatible properties, inertness, availability, and cost-efficient with the ability to imitate sound natural enamel [3,4]. Today, with the rising demand for metal-free restorations, 80.2% of crowns and fixed prostheses produced in the United States are all-ceramic restorations predominantly made of glass matrix [5,6]. However, a primary clinical concern is that ceramics are brittle with low fracture toughness that results in higher wear of the surface when exposed to the resultant shear forces of grinding [7,8,9,10,11]. In which case, the wear resistance of artificial dental materials in the oral environment was found to be critical for successful dental restoration. In this regard, many recent studies have been carried out to understand the oral lubrication mechanisms involved during the mastication process. From these the frictional forces arising from contact of two surfaces was found to be reduced by bio-lubricant saliva within the oral environment [12]. A better design of wear-resistant material in dental restorations and implants innovations might be obtained by applying lubrication to improve surface mechanical properties [13,14].

In the oral environment, saliva is essential in oral lubrication and primarily composed of water (99.5%), proteins (0.3%), and inorganic substances (0.2%). Among these, salivary proteins have been shown to act as aqueous boundary lubricants in physiology [15,16,17,18,19]. For example, both reflectometry and ellipsometry experiments have demonstrated that the salivary proteins in the oral cavity rapidly adsorb to any solid surface and form a complex proteinaceous film known as salivary pellicle [20,21]. One of the primary functions of the salivary pellicle is to lubricate the boundary surfaces, which subsequently reduces the wear of surfaces by diminishing the friction [22,23,24,25]. The literature revealed that this might be due to the formation of mono or multilayer of proteinaceous film depending on the proteins and the adsorbent surfaces involved, which can provide the aqueous boundary lubrication and reduce the friction [26]. Interestingly, the vast majority of the initial studies in this field were mainly concerned with the amount of the adsorbed proteins in terms of the protein thickness that may vary depending upon the pellicle’s location in the mouth [27,28]. Many research papers had been reported on this topic [29,30]. However, a fundamental understanding of the role of the structural changes, packing arrangement, and bioactivity of adsorbed proteins on artificial dental material in oral lubrication is still missing [29,30]. A possible reason may be due to the complexities introduced by protein–protein interactions (PPI) on the adsorption responses and lubrication behaviors of adsorbed proteins on the adsorbent surface.

The protein–protein interaction effect is defined to be proportional to the amount of the protein adsorbed on the surface (i.e., the areal density of the protein on the surface). PPI could be controlled for a given surface by varying the protein solution concentration from which the protein is adsorbed, with higher solution concentrations generally resulting in higher areal densities and more potent PPI effects at surface saturation [31,32,33]. The amount that an adsorbed protein will unfold and spread out on a surface is then influenced by PPI, with more substantial PPI effects that tend to sterically block further unfolding and spreading of an adsorbed protein because of the occupied space from the neighboring proteins.

The objective of the present study is to investigate the influence of protein–protein interaction effects on the structural changes and similar lubricating behaviors of adsorbed α-amylase on the dental ceramic material with glass as a model material which is predominantly used in the production of dental ceramics. α-Amylase is one of the predominant (40–50%) protein in human saliva that hydrolyzes the α-1,4 linkages of starch, glycogen, and other polysaccharides to glucose and maltose [34,35,36,37,38]. To address these issues, we laid out a tentative plan in which the α-amylase from varying solution concentrations was first adsorbed on the surface in order to vary the areal density of protein and the subsequent degree of PPI effects occurring within the adsorbed layer of protein. Then, the corresponding areal density and conformational structure of adsorbed proteins were monitored by measuring the shift in absorbance and circular dichroism (CD), respectively. Following this, the measurement of the friction coefficient was finally conducted to quantify the lubricating behaviors of the adsorbed protein layer on the glass surface. A friction coefficient is a dimensionless number that can be defined as the ratio of the friction force between two surfaces and the force holding them together [39]. Tribometers measure the friction coefficients in polymeric analogs of soft tissue within the mouth sliding/rolling against the hard surfaces of teeth or artificial dental material [28,40,41], with the friction coefficient reduced by introducing a lubricant film between two solid surfaces at a constant load [39]. In vitro tribological studies further suggested that the friction coefficient of the material surface was reduced when the wear resistance of the dental materials was improved [42,43,44].

Under these experimental conditions, with the constant internal protein stability and protein–surface interactions, we can thus quantify and isolate the influence of PPI effects on the structural and lubricating behaviors of the adsorbed α-amylase on a glass surface. Fundamental understanding of the salivary pellicle and the corresponding mechanisms of lubrication will enable the design and optimization of dental ceramic materials with improved wear resistance.

## 2. Materials and Methods

### 2.1. Protein and Material Surfaces

α-Amylase occurs widely in all living organisms and is the primary secretory protein constituent of saliva [45]. Soluble α-amylase (EC 3.2.1.1) from barley malt (HIMEDIA, West Chester, PA, USA) was selected for use in this study. The molecular weight determination using SDS-PAGE (sodium dodecyl sulfate-polyacrylamide gel electrophoresis), was found to be 50 kDa (See section S.1 on the supporting document). α-Amylase from different sources were found to bring about the same enzyme reaction, from which the hydrolysis of starch by the α-amylase of human saliva and malt yields identical split products [46]. The fused silica glass with a custom cut size (0.375′′ × 1.625′′ × 0.0625′′) was from Chuanfeng Enterprise Co., Ltd., Yilan, Taiwan. The glass surface composed of a silicon-oxygen network with a high density of hydroxyl groups was used to present the dental ceramic materials [47].

### 2.2. Material Surface Preparation and Characterization

#### 2.2.1. Preparation of Material Surfaces

Custom cut glass slides were procured to fit our CD cuvettes. The glass substrate used for adsorption studies were cleaned in a piranha solution (7:3 *v/v* H_2_SO_4_/H_2_O_2_) maintained at 50 °C by complete immersion for at least 30 min, followed by a basic wash (1:1:3 *v/v/v* NH_4_OH /H_2_O_2_/H_2_O) (Showa chemicals, Tokyo, Japan). To get rid of the impurities entirely from the glass surfaces, the above mentioned process was carried out more than one time. Subsequently, the glass slides were then subjected to ultrasonic cleaning with nano-pure water, followed by absolute (99.8%) ethanol (Honeywell Research Chemicals 24102-1L, Charlotte, NC, USA) rinsing. Later, a constant stream of nitrogen gas was blown over the slides to make them moisture-free and maintained at room temperature for consistency for further experiments.

#### 2.2.2. Characterization of Material Surfaces

Surface characterization was performed to determine the static air–water contact angle and atomic composition of the selected glass surfaces. Advancing contact angle was analyzed using a contact angle analyzer (100 SB, Sindatek Instruments co., Ltd., Taipei, Taiwan). It is one of the traditional methods used to determine the hydrophilicity of the material surfaces [48]. The quantitative elemental analysis was approached via energy-dispersive X-ray spectroscopy (X-Max^N^, Oxford Instruments, Oxford- shire, UK), which utilizes the characteristic spectrum of X-rays that is emitted by a sample, following initial excitation by the high-energy electron beam [49].

### 2.3. Protein Concentration and Adsorption Process

For measuring the bulk concentration of α-amylase in the solution, the absorbance of serial dilutions of 2.0 mg/mL stock protein solutions was made in a 96-well plate, with the stock protein concentrations being verified using a BCA assay (Pierce Biotechnology, Waltham, Massachusetts, USA) [50]. After complete reaction, the protein solution in the cuvette was read by an enzyme-linked immunosorbent assay (ELISA, BIOBASE Biotech Co., Ltd., Shandong, China) plate reader operated at 595 nm, and the optical density (O.D.) value was recorded to construct the standard calibration curve. By using the standard curve, the α-amylase concentration in the sample solutions could be calculated.

Protein adsorption was conducted at room temperature. α-Amylase powder was dissolved in nano-pure water at a different weight ratio to make different concentrations of protein solution, which includes 0.05, 0.1, 0.3, 0.5, 0.7, 1, 1.5, and 2 mg/mL respectively. After this, we immersed the procured glass slides in the protein solution with different concentrations for 2 h for complete adsorption. After 2 h of immersion, loosely adsorbed proteins on the surface during the adsorption process and proteins from the gas–liquid interfaces were removed by washing the surface with the solvent (nano-pure water) three times for further analysis.

### 2.4. Analysis of Adsorbed Proteins Using CD Spectroscopy

The structure of α-amylase in solution, the amount of protein adsorbed on the glass surface, and the subsequent adsorption-induced conformational changes of these adsorbed proteins were determined using CD spectroscopy. CD measurements were performed on a Jasco-810 spectrophotometer at room temperature over the wavelength range of 190 to 280 nm. The path length and the scan speed were 1 cm and 50 nm/min, respectively, with 0.2 nm resolution. Every spectrum was averaged three times.

The molar extinction coefficient of the protein (ε_205_) in solution at 205 nm was first determined by recording the background-corrected absorbance at this wavelength (*A_205_*) at varied solution concentrations verified as mentioned in Section 2.3 to determine the areal surface density of adsorbed protein. The molar extinction coefficient of the protein solution at 205 nm was obtained from the slope of the absorbance (*A_205_*) vs. (C_soln_*L) plot. The areal surface density of adsorbed protein (*Q_ads_*) was thus estimated by the following equation:

(1) $$Q_{ads}=\frac{A_{205}}{\varepsilon_{205}}$$

where *A*_205_ is the background-corrected absorbance at 205 nm, and *ε_205_* is the molar extinction coefficient that was determined for the protein solution at a wavelength of 205 nm. 

The background-corrected CD signals were converted to molar ellipticity (θ_mol_) using equation (2) and (3), respectively, to quantify the secondary structure in solution and adsorbed state of protein:

(2) $$\theta_{\mathrm{mol}}=\frac{\theta_{\mathrm{raw}}\times M}{1000\times C_{soln}\times L}$$

(3) $$\theta_{\mathrm{mol}}=\frac{\theta_{\mathrm{raw}}\times M}{10000\times Q_{ads}}$$

where θ_raw_ is the background corrected raw CD signal (See section S.2 in the supporting document), *M* is the mean residue molecular weight of 112 g/mol, *C_soln_* is the solution concentration of the protein (g/mL), *L* is the path length of the cuvette (cm), and *Q_ads_* is the surface density of adsorbed protein (g/cm^2^) [31].

From the obtained molar ellipticity (θ_mol_), the secondary structure content was estimated by the DichroWeb, an online data base [51,52].

### 2.5. Bioactivity Assay

#### 2.5.1. Preparation of DNS Reagent

Dinitrosalicylic acid (DNS) reagent used in this study was prepared by mixing 5 g of sodium hydroxide (Showa chemicals, Tokyo, Japan), 150 g of potassium sodium tartrate (Fisher chemicals), 1 g of crystalline phenol (Panrec Applichem, Darmstadt, Germany), 0.25 g of sodium hydrogen sulfite (Showa chemicals, Tokyo, Japan), and 5 g of 3,5-dinitrosalicylic acid (DNSA) (Panrec Applichem, Darmstadt, Germany) in a 500 mL of nano-pure water. The preparation method was based on the protocol mentioned in the previous study [53]. All chemicals were of analytical-reagent grade and were used as received.

#### 2.5.2. Determination of α-Amylase Activity

The α-amylase activity was quantitatively assayed by measuring the amount of reducing sugars released from soluble starch using the 3,5-dinitrosalicylic acid reagent method, as described by Miller (1959) [54]. Soluble starch stock solution (1% *w/v*) and α-amylase solution in the range of 0.1–8 μg were prepared by dissolving starch and α-amylase powders in nano-pure water (solvent) respectively and stored at 4 °C.

The activity assay was carried out separately by adding 0.5 mL of protein solution with varying amounts of protein concentration to the 7 mL of the soluble starch solution for the calibration of native protein activity. In contrast, glass slides with known concentrations of adsorbed protein were immersed in 7 mL of the soluble starch solution for the calibration of adsorbed protein activity followed by keeping in a water bath maintained at a constant temperature of 37 °C for 10 min. Later, 1 mL from the reacted solution of native and surface adsorbed α-amylase was taken in a separate glass test tubes and incubated in a boiling water bath with the addition of 2 mL of DNS reagent for 5 min. After the formation of color due to the reaction between reducing sugars and DNS reagent, the reacted solution was allowed to cool down to room temperature to measure the optical density of the adsorbed α-amylase using a UV-vis spectrophotometer at 540 nm. Each measurement procedure was performed three times. The specific activity of both native and adsorbed α-amylase was subsequently calculated by normalizing the obtained absorbance value (*A_540_*) by the total amount of protein in the solution and the complete protein adsorbed on the glass surface respectively. The relative bioactivity of the adsorbed α-amylase protein was calculated as mentioned in the below equation [55].

(4) $$\mathrm{Relative}\;\mathrm{bioactivity}\;\%=\frac{\mathrm{specific}\;\mathrm{activity}\;\mathrm{of}\;\mathrm{adsorbed}\;\mathrm{protein}}{\mathrm{specific}\;\mathrm{activity}\;\mathrm{of}\;\mathrm{native}\;\mathrm{protein}}\times 100$$

### 2.6. Friction Coefficient Determination of α-Amylase Covered Glass Surfaces

Friction tests were performed using CETR universal micro-tribometer-2 (UMT-2, Bruker, Campbell, CA, USA) with a unidirectional pin-on-disk designed model at room temperature. The glass slides adsorbed with α-amylase at varied bulk solution concentrations were mounted on the disk with pin adjusted perpendicular to it. In brief, the pin was rubbed against glass, which was locked on the lower stage disk (See Section S.3 in the Supplementary Materials). Nano-pure water was used as the lubricant in this test. The measurement program used here was the loading force of 50 milli-Newton (mN) with a rotational speed of 50 revolutions per minute (rpm). The rotation radius was about 10 mm, and the rotation time was 900 s. The glass surfaces were cleaned using 75% ethanol after each friction test. The friction coefficient was determined by the ratio of the sliding friction force (*F_x_*) measured by a tribometer to loading force (*F_z_*) applied, and the friction coefficient was calculated by averaging the values obtained from 800–900 s (more stable and consistent) for further comparison. Each condition was repeated three times.

### 2.7. Statistical Analysis

The results obtained from this study with *n* ≥ 3 were expressed as mean ± standard deviation (S.D.) and were studied statistically using a two-way analysis of variance (ANOVA) method. The statistical significance of differences between mean values for different protein concentration was analyzed using the Student’s t-test, with values of *p* < 0.05 being considered as statistically significant.

## 3. Results and Discussion

### 3.1. Surface Characterization

The results analyzed by the characterization techniques applied to the glass surfaces used in this study has been presented in Table 1. All of the measured values reported in Table 1 fall within the expected range [56,57,58], with the glass substrate exhibiting excellent surface hydrophilicity. Generally, surfaces with higher wettability show lower contact angles (< 90°), while the hydrophobic surface is indicated by the higher contact angles (> 90°) [59]. The glass surface thus has a strong potential to form hydrogen bonds with hydrogen bondable groups as well as ionic groups for electrostatic interactions.

### 3.2. The Areal Density of Adsorbed Protein and PPI Effects

**Figure 1:**
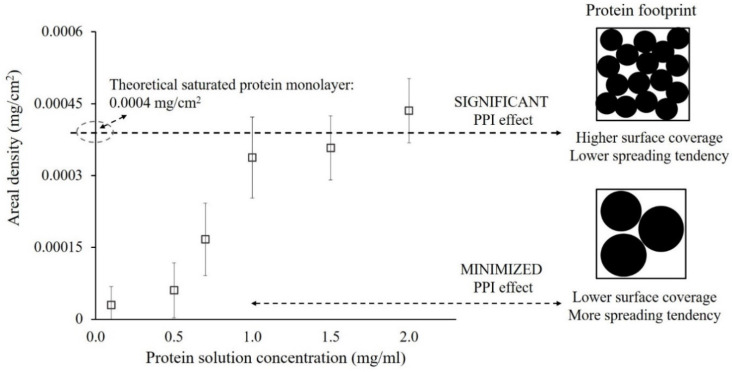
The adsorption capacity of the α-amylase protein on the glass surface at different concentrations. The error bars denote the mean ± SD for *n* = 3.

Figure 1 shows the bar graph of adsorption amount, which is represented as the weight of α-amylase adsorbed per unit glass surface area (mg/cm^2^) plotted against the different concentrations of α-amylase protein solutions. As shown, 2 h of exposure to the protein solution result in the widely distributed areal densities, generally increased with increasing solution concentration, thus provide a broad range of PPI effects within the adsorbed protein layer.

As indicated, the α-amylase interacted with a glass surface rapidly, with the adsorbed proteins unfolded and spread out over the surface because of the protein–surface interactions involved. The protein–surface interactions include electrostatic, hydrogen bonding, van der wall, hydrophobic, or dispersion interactions, which further increase their footprint on the surface [60,61]. The degree of spreading (i.e., conformational changes) of a protein over the surface will be determined by PPI effects with the constant internal protein stability and protein–surface interactions in this study. For example, when the protein solution concentration is increased due to the higher diffusion coefficient involved, there will be a subsequent increase in protein adsorption rate. From which more of the neighboring adsorption sites will be occupied by other adsorbing proteins, which will inhibit the spreading out of an adsorbed α-amylase on glass (i.e., when the PPI effect is significant).

On the other hand, when protein is adsorbed from lower solution concentration with a lower adsorption rate, the adsorbed protein tends to unfold and spread out before the occupation of neighboring sites by other proteins. From which the surface was found to be saturated with monolayer coverage of protein for each solution concentration. However, a lower amount of adsorbed protein was observed when it is from lower solution concentration. This saturation point can be theoretically calculated by assuming that the structure of the α-amylase is an intact sphere [62,63] with a dimension of about 5.97 × 10^−20^ cm^3^ to form the saturated monolayer at the areal adsorption density of 0.0004 mg/cm^2^ (See section S.4 in the supporting document).

α-Amylase is the major protein component present in the salivary pellicle [64], which adsorbs rapidly to the enamel and restorative material surfaces when exposed to saliva [65]. Once adsorbed, based on the PPI effects involved in this study, the glass surfaces were saturated with the adsorbed α-amylase at their native structure from high bulk solution concentration. In contrast, the unfolded α-amylase with significant conformational changes will saturate the glass surfaces at lower areal density observed because of bigger footprint when adsorbed from lower bulk solution concentration.

### 3.3. Influence of PPI Effects on Conformational Changes of Adsorbed Proteins

**Figure 2:**
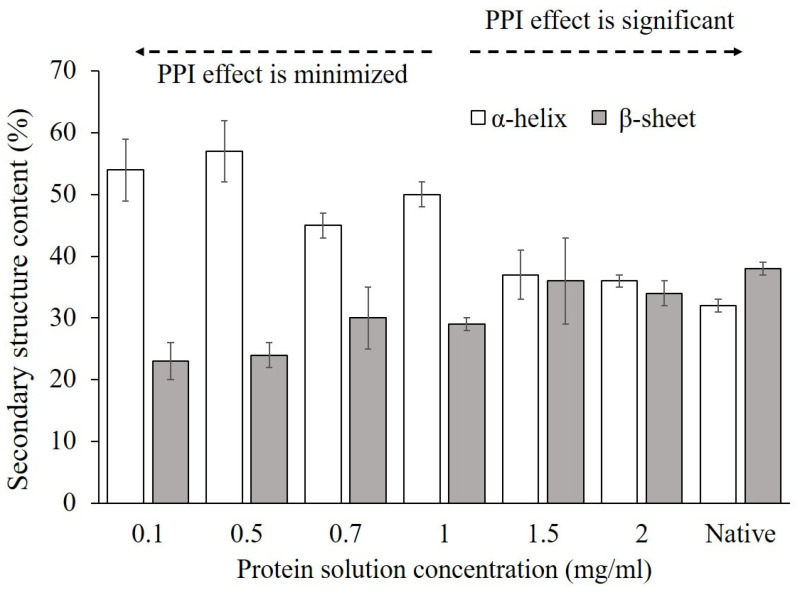
Secondary structure content (%) of α-amylase adsorbed onto the hydrophilic glass surface from different bulk solution concentrations. “Native” presents the native protein structure in solution. The error bars denote the mean ±SD for *n* = 3.

The secondary structure content obtained using the CD spectra of adsorbed α-amylase on the hydrophilic glass surface from varied bulk solution concentration, and native protein structure in solution is presented in Figure 2. As shown, the solution concentration from which α-amylase was adsorbed had a varied influence on the structures of adsorbed proteins on glass, with greater helicity and sheet structures being retained for adsorption from increased solution concentration representing significant PPI effect. In contrast, when the solution concentration was reduced, a substantial loss in sheet and an increase in helical structure of α-amylase upon adsorption was observed which is clearly due to the minimized PPI effect. The increased areal density of the adsorbed α-amylase layer on the glass is clearly shown to stabilize the protein against protein–surface interaction-induced unfolding.

Glass surface with a large density of hydrogen bondable and ionic groups may compete with the hydrogen bonds that stabilize the secondary structure of the protein, thus destabilize the secondary structures of adsorbed amylase [66,67], especially when PPI effect is minimized. On the other hand, the significant PPI effect tends to inhibit the conformational changes of the adsorbed α-amylase from the neighboring proteins, thus limiting the protein unfolding behaviors induced by hydrogen bonds from glass surface. 

It is now generally accepted that protein bioactivity is primarily determined by both of the conformation and accessibility of a protein’s bioactive site in an adsorbed protein layer [68]. To further explore if the PPI effects may affect the orientation changes of adsorbed proteins, the relationship between the conformation and bioactivity of adsorbed α-amylase on glass from varying protein solution concentrations is discussed in the next section.

### 3.4. Influence of PPI Effects on the Bioactivity of Adsorbed Proteins

**Figure 3:**
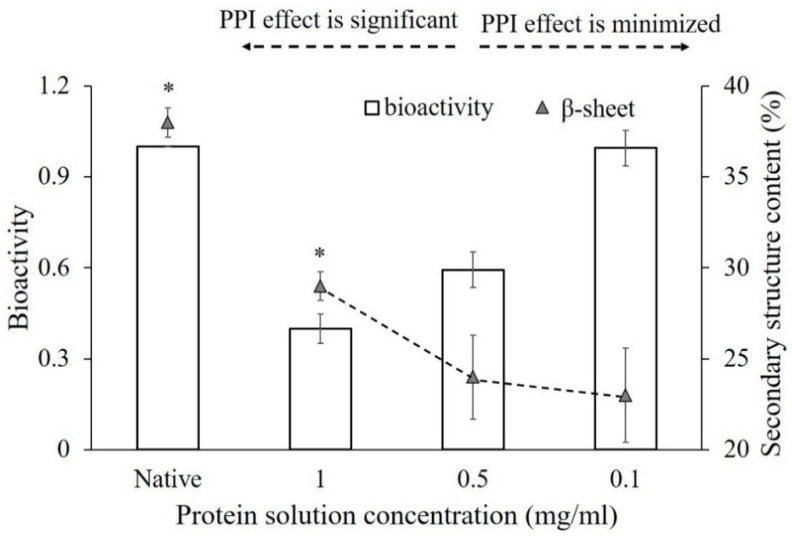
Comparison of the relative bioactivity of α-amylase adsorbed on the glass surface at different protein solution concentrations and the secondary structure change obtained. “Native” presents the native protein structure and relative bioactivity in solution. The error bars denote the mean ± SD for *n* = 3. *, *p* < 0.05 denote the significant differences in the bioactivity and % sheet content between native and high bulk protein concentration.

Figure 3 presents the relative bioactivities of an adsorbed α-amylase vs. its percent secondary structures on glass. As shown, α-amylase loses more than 40% of its bioactivity following adsorption from concentrated solution concentration when PPI is significant. From this an apparent correlation was observed between the retained structures of an adsorbed protein and its activity drop. These results suggest that, for an adsorbed α-amylase on glass, PPI effects primarily influence protein bioactivity by acting to block the accessibility of the bioactive site from neighbor proteins, which in turn helps preserve the protein structure.

On the other hand, the bioactive response of α-amylase vs. percent secondary structure changes was quite surprising when adsorbed from diluted protein solution concentration. As indicated in Figure 3, when the protein solution concentration was dropped from 0.5 to 0.1 mg/mL representing decreased areal density and PPI effects, an apparent increase in bioactivity was observed. In contrast, the protein secondary structure contents as the percentage sheet (%) structure were not significantly different. These results suggest that under low areal density conditions when PPI effects are minimized, the adsorption-induced structural changes of an adsorbed α-amylase on glass may still have its active sites exposed. From this an adsorbed α-amylase was able to retain its activity that was not significantly different than that of its native one on this type of surface, with protein activity reserved as protein structure unfolded.

**Figure 4:**
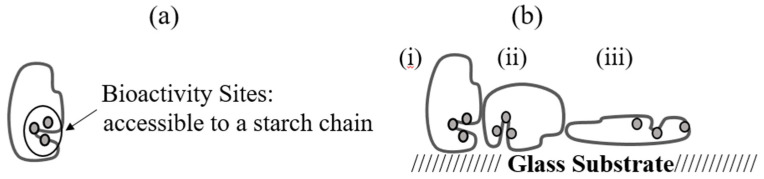
Illustration of the influence of various levels of protein–protein interactions (PPI) effects during the adsorption process on the bioactive sites of an α-amylase on the glass surface. (**a**) α-amylase in its native-state structure with its bioactive site accessible and intact for the hydrolytic reduction of starch. Their activity sites were contributed mainly by glutamate 233, aspartate 197, and aspartate 300 within the primary protein sequence working together to break the connection between two sugars in a starch chain [72,73]. (**b**) α-amylase adsorbed on a glass surface, with three types of interactions occurred namely: (i) The bioactive sites might be covered by the neighboring protein (high PPI effect), (ii) the bioactivity sites might be pointed toward the substrate surface (high or low PPI effect), and (iii) higher spreading of protein with the accessible bioactive sites retained and exposed toward the substrate in solution or with the adsorption-induced disruption of protein structures leading to the reduced connections between active sites (low PPI effect).

As mentioned in previous studies, a loss in protein activity on the glass surface was caused by the adsorption-induced conformational unfolding of the active sites [69]. However, it was also experimentally indicated that the activity of proteins after adsorption on the solid surfaces can still be reserved [70,71]. As shown in Figure 4b, the first two interactions will surely reduce the activity of the α-amylase as their active sites are blocked, which is believed to happen when the concentration of the solution is high, whereas the third type of interaction is found to occur at a low concentration where the protein structure will be changed. In which case, the protein activity may be retained due to the exposure of the bioactive sites after the structural changes or maybe reduced caused by the lost connections between the bioactive sites after conformational changes [69].

From this study, it is evident to see that the structure changes, packing arrangement, and bioactivity of adsorbed α-amylase on the glass surface tend to vary with the changed bulk protein solution concentrations applied. These data thus provide molecular-level insights into how PPI effects to influence the adsorption response of adsorbed proteins.

### 3.5. Influence of PPI Effects on Friction Coefficients

**Figure 5:**
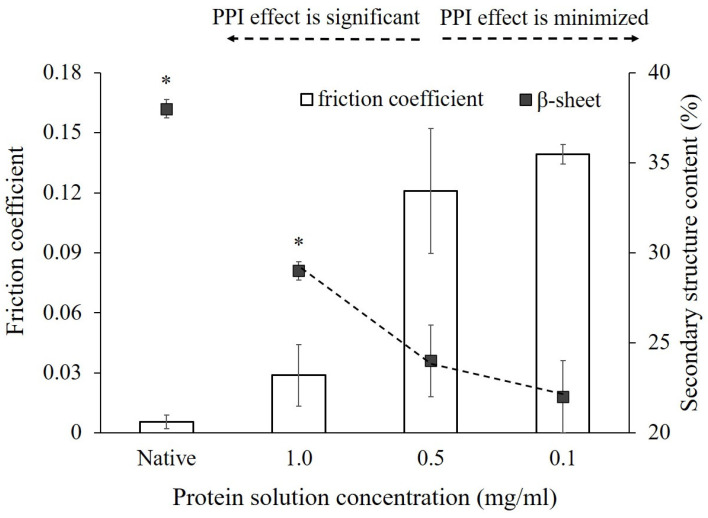
Comparison of friction coefficients exhibited on the surface at different protein concentrations and the subsequent structural changes. “Native” presents the native protein structure in solution and friction coefficient measured with no protein adsorbed on glass surface. The error bars denote the mean ± SD for *n* = 3. *, *p* < 0.05 denotes the significant differences in the friction coefficient and % sheet content between native and high bulk protein concentration.

Finally, the comparison between the secondary structure change induced during adsorption treatment at varying bulk protein solution concentrations and the coefficient of friction on the interacting surface was represented with a bar graph in Figure 5. Friction forces were measured using the unidirectional rotating pin-on-disk tribometer (polydimethylsiloxane (PDMS)-glass contact surfaces) between the upper PDMS stage operated in a normal force like the soft tissue in the mouth during mastication (i.e., 0.5N) [41,74] and the lower stage with the proteins-covered glass surface, representing the dental ceramic materials. As shown, we could see clearly that the coefficient of friction between the interacting surface was increasing with the increased secondary structural changes as presented by the reduced β-sheet content (%). As the increased friction coefficient has resulted from the increased friction force measured at the fixed normal force applied in this study, our data suggest that there might be the adhesive bridging interactions between PDMS and protein covered glass surface. In which case, a high-shear-strength layer might be formed from the adsorbed α-amylase unfolded with exposed functional groups subjected to tribological stress between two contact surfaces that ultimately leads to the high friction coefficient values observed. On the other hand, the adsorbed α-amylase exhibited the reduced friction coefficient values when more of its sheet structures were reserved when compared to that of a native protein, which may provide a lubricious, low-shear-strength, fluid film [28,75]. These results thus suggest that the friction observed between PDMS and glass is not just a result of the amount of proteins adsorbed on the glass surface [76] or the thickness of the adsorbed protein layer [27]. Other factors play a substantial role, such as possibly the influence of protein–surface interaction combined with the PPI effects on the protein’s conformational changes and the orientation on the surface and the subsequent accessibility of the functional groups. These combined interactions may provide additional interfacial adhesion with load-bearing abilities or drag force-dependent entrainment between PDMS and glass contact surfaces [28].

In this study, a preliminary plan has been laid out and applied to study the combined influence of protein–surface interactions and PPI effects on the adsorption responses of adsorbed α-amylase on glass surface, with the selected and simplified single protein model to provide molecular mechanisms by which the proteins covered glass may lead to the distinct lubricating behavior which in turn have an effect on the wear of the dental ceramic material surfaces. We believe that the theoretical assumptions made in this study may have some clinical relevance. For example, during the processing of liquid food or oral hygiene products in the oral cavity, the liquid and solid particles will be continuously pressed and mixed with saliva, which might affect the concentration of salivary proteins. From this, proteins will be adsorbed to dental glass surface under varying protein solution concentrations in saliva to saturate the surface to alter the PPI effects, leading to the various structural changes and lubricating behaviors of adsorbed proteins. The molecular-level explanation could then be clinically significant for the development of artificial saliva or saliva substitutes with appropriate protein content to provide better wear-protection of dental ceramic material.

However, tooth wear in the mouth is a complex multifactorial phenomenon involving chemical, physical, and mechanical processes [77]. In addition to PPI effect, the considerations of wear-resistant designs should include the influence of other inevitable factors. For example, the diet habit (types of food), oral-muscular forces during mastication, different hygiene measures on the friction, and loading forces involved in the oral cavity, will lead to different adsorption and lubricating behaviors of adsorbed salivary proteins as well. Therefore, to mimic the real clinical situation, the complete testing system considering more complex fluids, food types, and various loading forces applied simulating the chewing process, is required to explore the influence of the interaction between multiple salivary proteins in the boundary regime. In addition to the circular dichroism analysis conducted in this study, other techniques may provide additional molecular insights for investigating protein structural changes induced by adsorption behaviors. For example, the amino-acid labeling/mass spectrometry to assess adsorbed protein orientation and tertiary structure by monitoring adsorption-induced changes in residue solvent accessibility could be scheduled in our future studies.

## 4. Conclusions

In conclusion, we present an experimental approach to study the combined influence of protein–surface interactions and PPI effects on the conformational behavior and activity of adsorbed α-amylase on a glass surface, for a better understanding of the mechanisms behind the salivary lubrication performance.

The results from the structural studies suggest that PPI effects tend to stabilize the structure of α-amylase on glass with strong potential to form hydrogen bonding and electrostatic interactions with proteins. The bioactivity results indicate that PPI effects may influence adsorbed-state bioactivity by affecting the accessibility of the protein’s bioactive site. However, when PPI effect is minimized, the adsorption-induced structural changes would play a more direct role in the activity of an adsorbed α-amylase. In which case, its bioactivity increased in direct proportion to the degree of adsorption-induced disruption of the protein’s structure, which may lead to more exposure of the active sites of α-amylase on the glass surface. We speculate that this latter effect may be responsible for the lubricating performance of the unfolded protein layer on the glass surface by exposed functional groups of adsorbed α-amylase. We hope this comparative study with the understanding of the molecular-level effect of proteins on the tribology behavior of the hydrophilic glass surface can pave the way for a better design and development of the dental ceramic material with a high level of wear resistance in the oral environment.

## Figures and Tables

**Table 1:** Surface characterization: atomic composition and static contact angle analysis for the selected surface (mean ± SD, *n* = 3).

Surface Moiety	Si (%)	O (%)	Contact Angle (°)
GLASS	37.7 (2.0)	62.3 (2.0)	13 (2)

## Supplementary Materials

The following are available online at https://www.mdpi.com/2073-4360/12/8/1658/s1, Figure S1: SDS-PAGE of *α*-amylase from barley malt used in this study, Figure S2: CD spectra for Native and adsorbed *α*-amylase on glass surface when adsorbed from 0.1 mg/ml, 0.5 mg/ml and 1 mg/ml bulk solution concentrations, Figure S3: Schematic diagram of the friction testing machine, Table S1: The averaged values of coefficient of friction from the obtained values in the range of 800-900s.
