# Correction: Wei, Y.; et al. Quantify the Protein-Protein Interaction Effects on Adsorption Related Lubricating Behaviors of α-Amylase on a Glass Surface. *Polymers* 2020, *12*, 1658

**DOI:** 10.3390/polym13060925

**Published:** 2021-03-17

**Authors:** Nareshkumar Baskaran, You-Cheng Chang, Chia-Hua Chang, Shun-Kai Hung, Chuan-Tse Kao, Yang Wei

**Affiliations:** Department of Chemical Engineering and Biotechnology, National Taipei University of Technology, 1, Setion3, Zhongxiao East Road, Taipei 10608, Taiwan; adamnaresh1818@gmail.com (N.B.); youchengchang@gmail.com (Y.-C.C.); t104320088@ntut.org.tw (C.-H.C.); t105320036@ntut.org.tw (S.-K.H.); chuantsekao@gmail.com (C.-T.K.)

The authors wish to make the following corrections to this paper [1]: in the original version of our article, some of the data in Figures 1, 2, 3, and 5 were collected mistakenly from Lysozyme (Sigma, St. Louis, MO, USA), rather than α-amylase (EC 3.2.1.1) from barley malt (HIMEDIA, West Chester, PA, USA). We apologize for the original error. Figures (1, 2, 3, and 5) and corresponding descriptions should be updated to correct this oversight.

**1.** In Section 3.2. “The Areal Density of Adsorbed Protein and PPI effects” of the Results and Discussion on Page 6, Figure 1 was replaced with the updated data:

**Figure 1:**
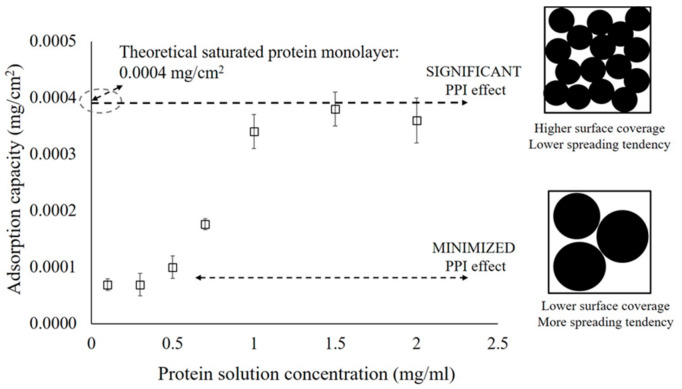
The adsorption capacity of the α-amylase protein on the glass surface at different concentrations. The error bars denote the mean ± SD for *n* = 3.

**2.** In Section 3.3. “Influence of PPI Effects on Conformational Changes of Adsorbed Proteins” of Results and Discussion on Page 7, Figure 2 was replaced with the updated data regarding the helical and random coil contents (%) of α-amylase.

In the first paragraph of Section 3.3, the second and the third sentences should be corrected to “As shown, the solution concentration from which α-amylase was adsorbed had a varied influence on the structures of adsorbed proteins on the glass. The greater helicity and less random coil structures (sheet structures in the original sentence) were retained for adsorption from increased solution concentration representing a significant PPI effect. In contrast, when the solution concentration was reduced, a substantial loss in helix and an increase in the random coil structures (loss in sheet and an increase in helical structure in the original publication) of α-amylase upon adsorption were observed clearly due to the minimized PPI effect.”

**Figure 2:**
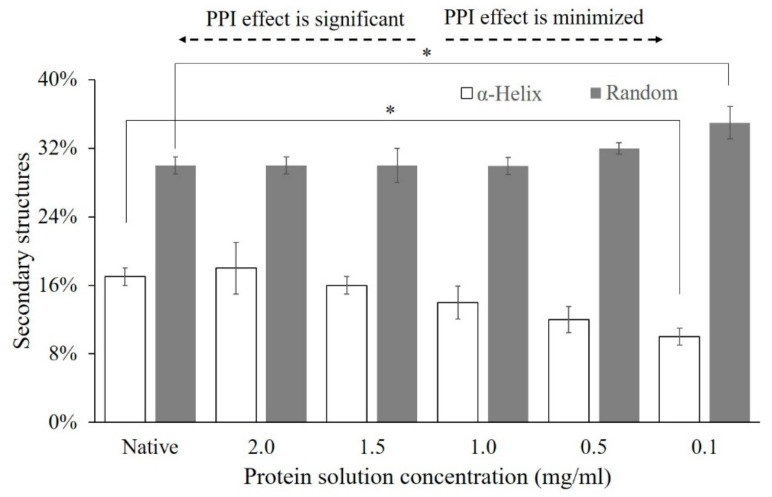
Secondary structure content (%) of α-amylase adsorbed onto the hydrophilic glass surface from different bulk solution concentrations. “Native” presents the native protein structure in the solution. The error bars denote the mean ± SD for *n* = 3. * represents the significant difference with *p* < 0.05.

**3.** In Section 3.4. “Influence of PPI Effects on the Bioactivity of Adsorbed Proteins” of Results and Discussion on Page 8, Figure 3 was replaced with the updated data regarding the helical structures of α-amylase.

Line 5 in the secondary paragraph of Section 3.4 should be corrected to “In contrast, the protein secondary structure contents as the helical contents (%) (percentage sheet (%) in the original publication) were not significantly different.”

**Figure 3:**
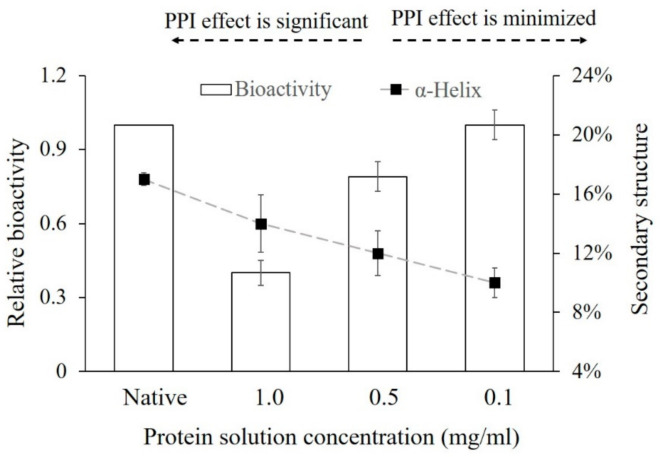
Comparing the relative bioactivity of α-amylase adsorbed on the glass surface at different protein solution concentrations and the secondary structure change (α-helix (%)) obtained. “Native” presents the native protein structure and relative bioactivity in the solution. The error bars denote the mean ± SD for *n* = 3.

**4.** In Section 3.5. “Influence of PPI Effects on Friction Coefficients” of Results and Discussion on Page 10, Figure 5 was replaced with the updated data regarding the helical structures of α-amylase.

Line 7 to Line 9 on page 9 in the first paragraph of Section 3.5 should be corrected to “We could see clearly that the coefficient of friction between the interacting surface was increasing with the increased secondary structural changes as presented by the reduced helical content (%).”

Line 14 to 16 on page 10 in the first paragraph of Section 3.5 should be corrected to “The adsorbed α-amylase exhibited reduced friction coefficient values when more of its secondary structures (sheet structures in the original publication) were reserved when compared to that of a native protein. The adsorbed protein layer may provide a lubricious, low-shear-strength, fluid film.”

**Figure 5:**
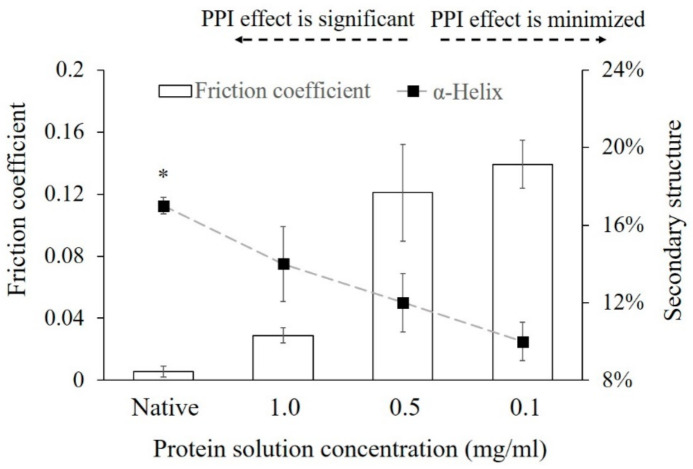
Comparison of friction coefficients exhibited on the surface at different protein concentrations and the subsequent structural changes (α-helix (%)). “Native” presents the native protein structure in solution and friction coefficient measured with no protein adsorbed on the glass surface. The error bars denote the mean ± SD for *n* = 3. * represents the significant difference in helix structure and friction coefficient with *p* < 0.05.

The authors apologize for any inconvenience caused and state that other scientific information is unaffected. The original article has been updated.
