# Supplementary material for: Quantify the Protein–Protein Interaction Effects on Adsorption Related Lubricating Behaviors of α-Amylase on a Glass Surface
Source: Polymers (Basel). 2020 Jul 25;12(8):1658. doi: 10.3390/polym12081658 (PMC7463787; doi:10.3390/polym12081658)
Supplement: Supplementary file 1 [file polymers-12-01658-s001.pdf]

Quantify the Protein-Protein Interaction Effects on Adsorption related Lubricating Behaviors of α-Amylase on a Glass Surface

Nareshkumar Baskaran[1], You-Cheng Chang[1], Chia-Hua Chang[1], Shun-Kai Huang[1], Chuan-Tse Kao[1], and Yang Wei[1]*

[1]Department of Chemical Engineering and Biotechnology, National Taipei University of Technology, 1, Sec. 3, Zhongxiao E. Rd., Taipei 10608, Taiwan

This supporting information contains (*i*) molecular weight determination, (*ii*) raw CD spectral data of native and adsorbed α- amylase responses under varying PPI conditions effect, (*iii*) friction test using pin-on-disk tribometer, and *(iv)* theoretical calculation of the monolayer adsorption capacity.

S.1 Determination of the molecular weight

The molecular weight determination of α- amylase using sodium dodecyl sulfate-polyacrylamide gel electrophoresis (SDS-PAGE) was performed according to a previous study[1]. The chemicals required for the analysis was prepared in an appropriate quantity. For the formation of separating gel, 1.5 M Tris was made and the pH value was adjusted to 8.8. For the stacking gel preparation, 1 M Tris was prepared, and the pH value was adjusted to 6.8. The concentration of the gel was determined according to the molecular weight of the protein sample. 1 mg/ml of α- amylase solution was mixed with 4 X SDS loading buffer (2% SDS; 10% glycerol; 0.5% bromophenol blue and 0.5M Tris-HCl, pH 6.8) and kept in boiling water (100°C) for 10 min. Then ten µl of the sample was loaded to each lane of 5 to 15% Bis-Tris gradient gels and separated at 50A and 120 V/gel using Mini Protean Tetra Cell units (Bio-Rad Laboratories, Inc., Richmond, CA). Following the separation, the gel was stained with Coomassie Brilliant Blue G-250 (Bioshop Canada Inc., Burlington, ON, Canada) for 20 minutes and then destained twice with acetic acid (PanReac AppliChem GmbH, Ottoweg, Darmstadt, Germany).

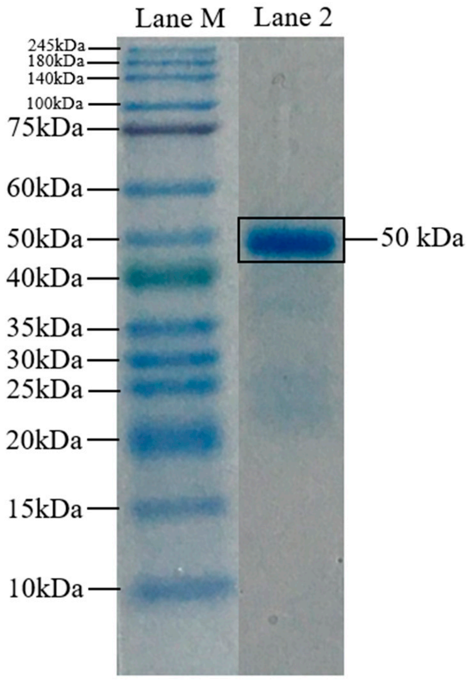

Figure S1. SDS-PAGE of α-amylase from barley malt used in this study. Lane M and Lane 2 denotes the marker and α-amylase loaded lane, respectively.

From the figure, it is more apparent that the molecular weight of the α-amylase used in this study is about 50 kDa. This molecular weight of α-amylase determined was used as the mass of each α-amylase protein for the theoretical calculation of the single-layer protein adsorption capacity described in detail in the section S.4.

S.2 Raw CD spectral data of native and adsorbed α- amylase responses under varying PPI conditions effect

Fig. S2. Illustrates the sample CD spectra for the native (i.e., α-amylase protein solution) and adsorbed proteins on the glass surface when adsorbed from 0.1 mg/ml, 0.5 mg/ml and 1 mg/ml bulk solution concentrations for an adsorption time of 2 hours and then equilibrated in the nano-pure water for another 2 hours. This obtained molar ellipticity (θ_{mol}) was uploaded on the DichroWeb, an online database to know the percentage of helical and sheet content [2, 3].

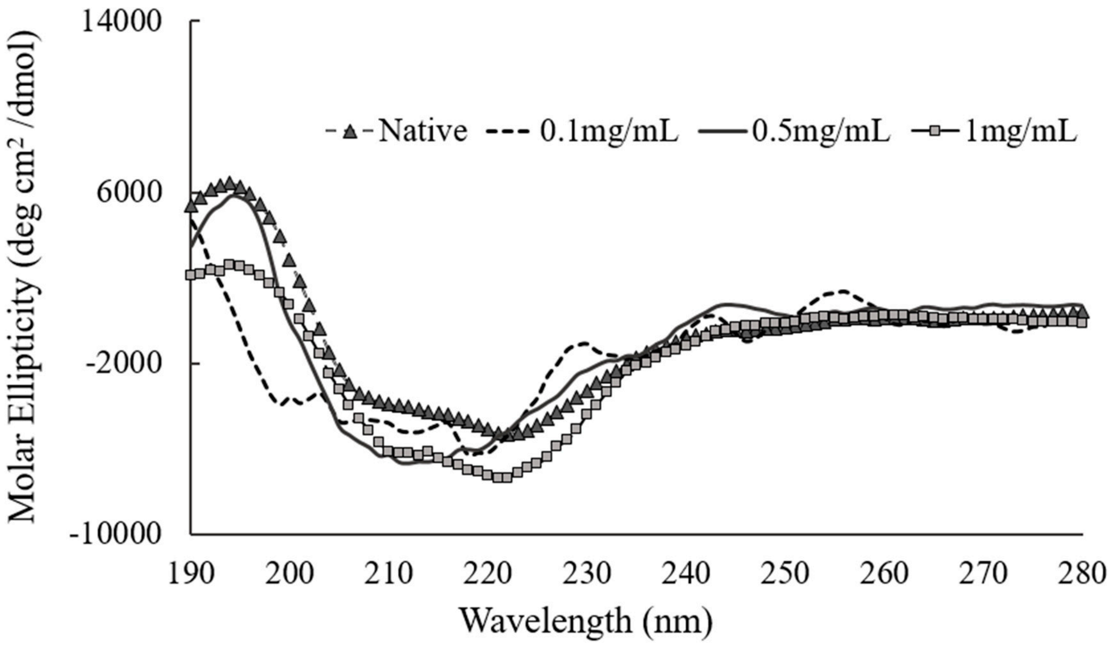

Figure S2. CD spectra for Native and adsorbed α-amylase on glass surface when adsorbed from 0.1 mg/ml, 0.5 mg/ml and 1 mg/ml bulk solution concentrations. (Average of 3 spectra). "Native" denotes the native α-amylase protein in solution.

S.3 Friction test using a pin-on-disk tribometer

A schematic of the experimental pin-on-disk tribometer was shown in Fig. S3. The upper stage with a pin was made of PDMS, a soft oral-mimetic surface[4]. The pin was rubbed against the glass with adsorbed α-amylase locked in the lower stage with a force of 0.5 N, which is the critical surface tension for saliva-coated tooth surfaces as reported in the previous studies[5]. The coefficient of friction was calculated by the ratio of the sliding friction force (F_x) to the forward loading force (F_z), as mention in equation (S1).

$$Coefficient\ of\ friction, COF = \frac{F_X}{F_Z} \qquad \text{(S1)}$$

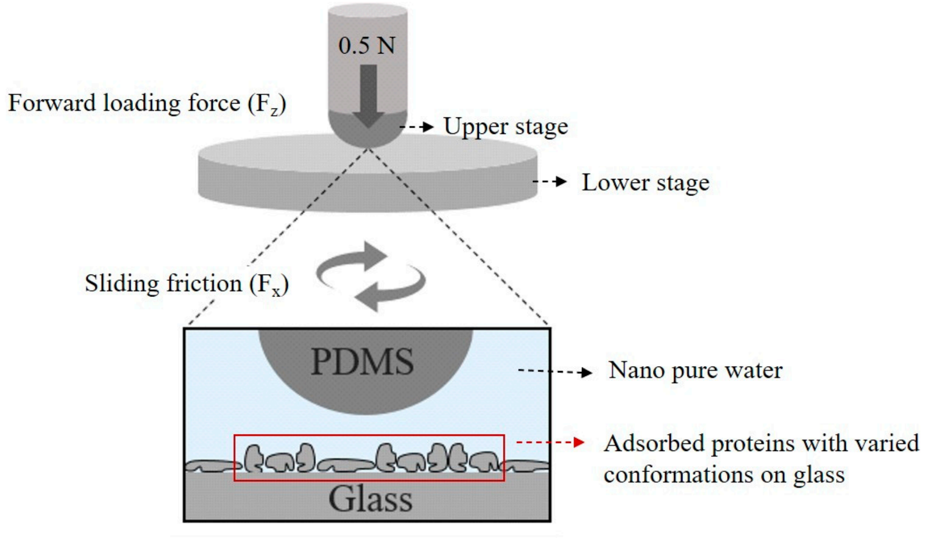

Figure S3. Schematic diagram of the friction testing machine.

Table S1. The averaged values of coefficient of friction from the obtained value in the range of 800-900s

α-Amylase protein concentration (mg/ml)	Obtained COF	Averaged COF
Native	0.009546	0.0055
Native	0.002883	
Native	0.004164	
0.1	0.13407	0.1393
0.1	0.127179	
0.1	0.156576	
0.5	0.098456	0.121
0.5	0.107971	
0.5	0.156606	
1	0.034066	0.0288
1	0.028057	
1	0.024344	

S.4 Theoretical calculation of the single-layer adsorption capacity

The monolayer adsorption capacity can be theoretically calculated by assuming that the structure of the α-amylase adsorbed on the glass surface is a spherical footprint. Previous studies have proved that the average density of the proteins with a molecular weight of around 40-50 kDa was about 1.41g/cm^3[6]. Spherical volume (v) and surface area (A) of the α-amylase, as shown in equation (S2) and (S3) respectively, was used as per our assumption, where radius (r) can be calculated using (Equation S2).

$$v = \frac{4}{3}\pi r^3 \quad (S2)$$

$$A = \pi r^2 \quad (S3)$$

$$M = \frac{1}{\pi r2} X \frac{1}{6.02*10^{\wedge}20} X\, 50000 \quad (S4)$$

$$V = 50000\, X \frac{1}{6.02*10^{\wedge}20}\, X \frac{1}{1.41} \quad (S5)$$

Theoretical protein mass per unit area (M) (i.e., single-layer adsorption capacity) and the volume of each protein (V) (i.e., mass/density) adsorbed was calculated as 0.0004 mg/cm^3 and $5.97x10^{-20}cm^3$ using the equation (S4) & (S5) respectively which was derived from the previous paper[7].

References:

[1] U.K. Laemmli, Cleavage of structural proteins during the assembly of the head of bacteriophage T4, nature 227(5259) (1970) 680-685.

[2] L. Whitmore, B.A. Wallace, DICHROWEB, an online server for protein secondary structure analyses from circular dichroism spectroscopic data, Nucleic acids research 32(Web Server issue) (2004) W668-73.

[3] L. Whitmore, B.A. Wallace, Protein secondary structure analyses from circular dichroism spectroscopy: Methods and reference databases, Biopolymers 89(5) (2008) 392-400.

[4] A. Sarkar, E. Andablo-Reyes, M. Bryant, D. Dowson, A. Neville, Lubrication of soft oral surfaces, Current Opinion in Colloid & Interface Science 39 (2019) 61-75.

[5] C.E. CHRISTERSSON, R.G. DUNFORD, P.O.J. GLANTZ, R.E. BAIER, Effect of critical surface tension on retention of oral microorganisms, European journal of oral sciences 97(3) (1989) 247-256.

[6] H. Fischer, I. Polikarpov, A.F. Craievich, Average protein density is a molecular-weight-dependent function, Protein Science 13(10) (2004) 2825-2828.

[7] M. Rabe, D. Verdes, S. Seeger, Understanding protein adsorption phenomena at solid surfaces, Advances in colloid and interface science 162(1-2) (2011) 87-106.
